# Line Strengths and Lifetimes of Levels in Neutral Uranium

**DOI:** 10.6028/jres.080A.002

**Published:** 1976-02-01

**Authors:** Charles H. Corliss

**Affiliations:** Institute for Basic Standards, National Bureau of Standards, Washington, D.C. 20234

**Keywords:** Lifetimes in U I, oscillator strengths for U I, transition probabilities for U I, uranium spectrum

## Abstract

Relative intensities of 549 U I lines observed in a dc copper arc are used to derive transition probabilities and oscillator strengths. Upper limits to lifetimes for 65 levels in neutral uranium atoms are determined.

## 1. Introduction

Separation of isotopes by excitation of excited states through absorption of laser radiation is a relatively new development that has particular application in separation of uranium isotopes. Knowledge of approximate values for oscillator strengths of U I lines and lifetimes of corresponding upper levels is useful in that work. This paper reports such values for 549 lines and 65 levels of U I.

## 2. Derivation of Oscillator Strengths

The observations from which the oscillator strengths reported here are derived were taken from the NBS Tables of Spectral-Line Intensities by Meggers, Corliss, and Scribner [1961]. Those intensities were measured in a 10 A dc arc between copper electrodes containing one atom of uranium for every 1000 atoms of copper. In 1962 Corliss and Bozman published oscillator strengths for 326 lines of U I. They used an arc temperature of 5100 K to obtain a relative scale which was then adjusted to an absolute scale by comparison with other elements for which absolute oscillator strengths were then known.

In the 13 years that have elapsed since then, much more accurate work on determination of oscillator strengths has been reported. The number of papers published on the subject now amounts to nearly 2400, whereas in 1962 it was only 600. This greatly expanded and also improved knowledge of oscillator strengths has given us a better understanding of the population distribution of excited levels of atoms in the copper arc of Meggers, Corliss, and Scribner. According to the study made by Corliss in 1962, the best oscillator strengths then available indicated that the level population of atoms in our copper arc declined by a factor of 17 for every 10 000 cm^−1^ rise in level value. Recent measurements of oscillator strengths show that the arc is in fact hotter, so that the corresponding decline in level populations is more nearly a factor of 10. This factor of 10 is used in the present work to calculate relative oscillator strengths in U I.

Since about 1964 there have been many measurements of lifetimes of atomic levels by means of beam-foil spectroscopy and since 1956, by the method of delayed coincidence. These new measurements make conversion of relative scales to absolute scales much more certain than previously. The method of delayed coincidence has been used recently by Klose [1975] to measure the lifetime of the U I level at 27887 cm^−1^ above the ground state. I have equated his value for this level, 7.3 ns, to the reciprocal of the sum of the transition probabilities of the two principal transitions (at 3584.88 and 4620.23 Å) from the level to adjust the relative values to the absolute scale.

## 3. Accuracy of Results

The standard deviation of an individual determination of the intensity from Meggers, Corliss and Scribner is about 32 percent. Klose’s uncertainty in the lifetime is about 15 percent. These two uncertainties affect all of the normalized transition probabilities equally. The log of the uncertainty in the value adopted for the rate of decline of population of levels of U I in the arc plasma is estimated to be ± 0.1 for every 10 000 cm^−1^ that the upper level departs from the level of normalization at 27887 cm^−1^. A line with an upper level separated about 5000 cm^−1^, for example, from that level would have an uncertainty in the log *gf* of ± 0.05 arising from the population uncertainty. The corresponding uncertainties of 0.12 in the intensity and 0.06 in the lifetime when combined quadratically with the chosen example of uncertainty in population brings the total error to 0.14 (an error of 38 percent in this case. More than half of the U I lines reported here arise from levels that lie within 5000 cm^−1^ of the normalized level.

Recently Voigt [1975] has measured oscillator strengths for 22 lines of U I. His results are systematically different from the values reported here. His values are smaller than mine below 4240 Å and larger at longer wavelengths. It can also be said that his values are smaller than mine for upper energy levels above 24 000 cm^−1^ and larger at lower energy levels. Whether the wavelength or the energy level is significant for the discrepancy is not clear.

The uncertainties in the level lifetimes calculated from the transition probabilities can be determined from the uncertainties of these transition probabilities but there remains an additional error arising from unobserved transitions from each level not included in the summation. The existence of this error requires that the lifetimes be regarded as upper limits. If, however, calculation of lifetimes is restricted to only those levels involving relatively strong lines, the error due to faint lines will be small. In this paper we have restricted the calculation of lifetimes to upper levels for which the sum of the intensities of downward transitions given in Meggers, Corliss, and Scribner is 20 or larger on their scale. Since the faintest observed lines are between 0.3 and 7 on that scale, depending on the spectral region, there is some assurance that the given upper limit will not be far above the correct lifetime.

As an example, we show below the lifetimes for three levels from unpublished measurements obtained by a method of laser excitation and subsequent isotope separation by Janes, Itzkan, Pike, Levy, and Levin [1975] compared with our intensity summations and corresponding derived lifetimes. Note that the lifetime for the case where the ΣI > 20 is in much better agreement than the cases involving only faint lines.

**Table:** 

Level cm^−1^	Lifetime *ns* (J, I, P, L&L)	ΣI	Lifetime *ns* (Corliss)
23572	60	33	66
23433	155	4	490
23212	170	4.6	320

This limited comparison tends to support our reasoning.

## 4. Tables of Results

Table 1 lists the wavelength in Angstroms; the lower and upper levels in cm^−1^; *gA*, the weighted transition probability in units of 10^8^ transitions per second; *gf*, the weighted oscillator strength, and log *gf* (base ten). The wavelengths are from Harrison’s MIT Wavelength Tables [1939] or from unpublished measurements of Kiess, Humphreys, and Laun [1946]. The levels are from a thesis by Guyon [1972].

Table 2 lists the upper levels in cm^−1^, the *J*-value of the level, the statistical weight (2*J* + 1), the upper limit of the lifetime in nanoseconds (10^−9^ s), and the number of transitions in the summation. All of these levels are of even parity.

## Figures and Tables

**Table 1:** Transition probabilities for lines of U I

Wavelength Å	Energy levels cm^−1^	gA 10^8^/s	gf	log gf
2855.96	0–35004	3.2	.39	−.41
2926.13	0–34164	1.8	.23	−.64
2951.93	620–34486	2.4	.32	−.50
2998.36	0–33341	1.9	.25	−.59
3027.66	620–33640	4.1	.56	−.25
3048.64	620–33412	4.7	.65	−.19
3114.54	0–32098	2.1	.30	−.52
3147.09	620–32386	3.5	.52	−.28
3214.70	0–31098	.73	.11	−.95
3263.12	0–30637	1.1	.18	−.75
3293.56	0–30353	.63	.10	−.99
3319.21	3868–33987	2.9	.48	−.32
3345.56	0–29881	.76	.13	−.89
3345.89	620–30499	1.3	.22	−.65
3360.00	0–29753	1.1	.19	−.72
3368.98	0–29673	.55	.094	−1.03
3378.20	4453–34046	1.5	.26	−.59
3390.39	620–30107	6.1	1.1	.02
3390.97	3800–33282	2.1	.36	−.44
3395.52	0–29442	2.1	.36	−.44
3397.19	4275–33703	1.4	.24	−.62
3405.72	3800–33154	1.6	.29	−.54
3407.87	3800–33136	2.5	.43	−.37
3414.32	4453–33733	1.4	.25	−.61
3418.39	620–29866	.58	.10	−.99
3430.18	3868–33013	.80	.14	−.85
3431.14	4276–33412	2.6	.46	−.33
3433.90	7645–36758	5.7	1.0	.00
3434.61	0–29107	.82	.15	−.84
3435.20	3800–32902	2.3	.42	−.38
3435.49	0–29099	3.9	.70	−.16
3442.95	0–29036	1.8	.32	−.50
3448.78	0–28987	.96	.17	−.77
3458.17	3800–32709	3.8	.68	−.17
3459.92	0–28894	2.4	.42	−.37
3462.21	0–28875	2.0	.37	−.44
3463.54	620–29484	3.4	.62	−.21
3466.30	0–28840	4.1	.73	−.14
3469.49	3800–32615	2.2	.40	−.40
3469.78	0–28811	.78	.14	−.85
3473.43	3800–32582	4.8	.86	−.06
3480.36	5762–34486	8.5	1.5	.19
3489.37	0–28650	9.8	1.8	.25
3493.99	620–29233	2.4	.44	−.36
3497.26	3800–32386	2.1	.39	−.41
3500.07	0–28563	3.8	.71	−.15
3501.00	4453–33008	1.2	.23	−.65
3502.24	3868–32413	3.6	.66	−.18
3504.00	3800–32331	4.6	.84	−.08
3504.93	0–28523	.88	.16	−.79
3507.05	620–29126	1.7	.31	−.51
3507.34	0–28504	4.7	.86	−.06
3511.44	0–28470	1.5	.27	−.57
3513.68	620–29072	2.2	.40	−.40
3514.61	0–28444	9.4	1.7	.24
3516.85	3868–32294	2.1	.39	−.41
3522.67	3800–32180	2.0	.38	−.42
3525.65	0–28355	1.4	.26	−.58
3531.64	620–28927	1.6	.30	−.52
3534.33	0–28286	1.8	.34	−.47
3535.84	620–28894	.48	.091	−1.04
3537.28	0–28262	1.4	.26	−.58
3538.23	620–28875	.80	.15	−.82
3539.65	3800–32044	3.3	.62	−.21
3542.57	4276–32496	4.8	.90	−.05
3543.73	7326–35536	3.7	.69	−.16
3545.44	4275–32472	3.7	.69	−.16
3545.67	4453–32648	3.8	.72	−.14
3546.13	620–28811	1.6	.30	−.52
3548.62	4275–32447	2.2	.41	−.38
3550.17	5762–33921	3.1	.58	−.24
3552.95	4453–32590	1.1	.21	−.67
3553.44	3800–31934	1.9	.37	−.43
3555.32	0–28119	3.8	.72	−.14
3557.84	0–28099	2.2	.41	−.39
3560.31	5991–34070	3.2	.60	−.22
3561.41	3800–31871	4.2	.79	−.10
3561.80	0–28067	6.4	1.2	.09
3563.66	0–28053	2.1	.41	−.39
3564.18	620–28669	.77	.15	−.83
3565.05	4276–32318	2.1	.41	−.39
3566.60	620–28650	15.	2.8	.44
3571.16	620–28614	.92	.18	−.76
3574.11	7005–34976	5.2	1.0	.00
3574.76	0–27966	1.7	.33	−.48
3577.92	0–27941	2.0	.38	−.42
3578.33	0–27938	1.3	.25	−.60
3580.25	620–28543	1.2	.23	−.64
3582.62	4275–32180	2.8	.53	−.27
3584.88	0–27887	17.	3.3	.51
3585.84	620–28499	1.5	.29	−.54
3587.78	4453–32318	1.8	.34	−.46
3589.66	620–28470	1.5	.29	−.54
3589.79	3800–31649	3.1	.59	−.23
3591.74	620–28454	2.4	.46	−.34
3592.97	620–28444	.74	.14	−.84
3593.20	4276–32098	3.4	.66	−.18
3593.68	5762–33580	2.4	.46	−.34
3600.29	620–28387	.88	.17	−.77
3601.19	8118–35879	2.4	.47	−.33
3602.48	3801–31552	1.8	.35	−.45
3603.36	0–27744	.76	.15	−.83
3603.74	4276–32017	1.7	.33	−.49
3605.28	0–27729	2.4	.47	−.33
3610.69	4453–32141	2.1	.41	−.39
3611.40	0–27682	.37	.073	−1.14
3615.54	0–27650	.74	.15	−.84
3616.33	3801–31445	3.8	.75	−.12
3617.49	7103–34739	1.9	.37	−.43
3617.62	3800–31435	.88	.17	−.76
3620.08	0–27616	1.6	.31	−.50
3622.04	0–27600	.37	.072	−1.14
3622.70	4275–31871	4.2	.83	−.08
3634.56	3868–31374	1.5	.29	−.54
3635.30	3801–31301	1.7	.34	−.47
3637.51	5991–33474	3.3	.66	−.18
3638.20	3801–31279	14.	2.7	.43
3638.65	8118–35593	5.4	1.1	.03
3642.44	4275–31722	2.8	.57	−.25
3644.24	620–28053	3.3	.65	−.18
3649.58	5762–33154	2.2	.44	−.36
3651.53	3800–31178	11.	2.1	.33
3652.07	4275–31649	8.7	1.7	.24
3653.21	3801–31166	2.2	.45	−.35
3654.89	3868–31221	2.3	.45	−.34
3659.16	620–27941	7.4	1.5	.17
3659.59	620–27938	1.3	.27	−.57
3667.13	0–27261	.69	.14	−.86
3669.18	5762–33008	3.0	.60	−.22
3670.53	7645–34881	9.2	1.9	.27
3674.13	620–27829	1.4	.29	−.53
3677.39	3801–30986	4.1	.82	−.09
3679.38	620–27791	1.8	.37	−.43
3680.88	4275–31435	4.2	.85	−.07
3682.46	0–27148	.90	.18	−.74
3685.78	620–27744	1.4	.29	−.54
3697.13	3800–30841	3.7	.75	−.12
3702.62	4276–31276	4.9	1.0	.01
3703.27	620–27616	2.1	.44	−.36
3704.09	7326–34315	9.9	2.0	.31
3707.29	3800–30767	4.4	.91	−.04
3707.95	3868–30829	2.1	.43	−.36
3709.87	5762–32709	3.2	.67	−.18
3713.56	0–26921	2.8	.58	−.24
3715.47	4275–31182	4.9	1.0	.00
3716.14	3800–30702	2.0	.42	−.37
3719.29	620–27499	.99	.20	−.69
3720.39	3800–30672	4.3	.90	−.05
3722.68	7191–34046	9.4	1.9	.29
3731.45	0–26792	.74	.15	−.81
3731.77	7645–34434	4.8	1.0	.00
3732.26	3801–30587	4.3	.89	−.05
3733.58	6249–33025	7.4	1.6	.19
3742.35	7005–33719	7.2	1.5	.18
3751.18	5762–32412	17.	3.5	.55
3751.72	620–27267	2.1	.45	−.35
3758.36	4276–30876	9.7	2.1	.31
3763.27	7006–33571	12.	2.5	.41
3765.35	0–26550	2.0	.43	−.37
3766.89	4453–30993	8.9	1.9	.28
3773.44	6249–32742	17.	3.5	.55
3776.48	4275–30747	5.8	1.2	.09
3781.75	7645–34080	5.1	1.1	.04
3786.84	6249–32648	7.3	1.6	.20
3788.16	5991–32381	5.4	1.2	.06
3792.41	4275–30636	2.3	.50	−.30
3793.28	6249–32604	11.	2.4	.39
3796.20	3868–30203	4.2	.91	−.04
3797.77	11308–37631	18.	3.9	.59
3798.84	11308–37624	23.	5.0	.69
3801.15	620–26921	2.0	.43	−.37
3808.93	6249–32496	11.	2.4	.38
3810.10	3868–30107	1.6	.35	−.46
3812.00	0–26226	13.	2.9	.46
3818.06	5762–31945	4.2	.92	−.04
3819.25	4276–30451	2.2	.49	−.31
3821.95	3801–29958	4.0	.88	−.06
3822.35	10347–36501	8.9	2.0	.29
3829.79	0–26104	.83	.18	−.74
3831.86	4275–30365	4.4	.97	−.01
3835.22	0–26066	.82	.18	−.74
3839.62	3801–29838	20.	4.3	.64
3846.24	7005–32997	4.0	.89	−.05
3846.55	3801–29791	3.9	.86	−.07
3854.22	0–25938	4.1	.92	−.04
3863.09	3868–29747	1.9	.43	−.37
3871.04	0–25826	9.6	2.2	.33
3876.13	0–25792	1.6	.35	−.45
3879.53	4453–30222	4.3	.97	−.01
3894.12	0–25672	3.1	.70	−.16
3899.27	4275–29914	4.0	.92	−.04
3906.46	3868–29459	5.7	1.3	.11
3908.32	4453–30032	2.1	.48	−.32
3917.25	8119–33640	9.5	2.2	.34
3926.22	0–25463	2.0	.45	−.34
3928.83	0–25445	.73	.17	−.77
3943.82	0–25349	7.2	1.7	.22
3948.45	0–25319	1.7	.41	−.39
3951.48	4453–29753	1.5	.36	−.44
3955.38	7103–32378	7.2	1.7	.23
3959.20	7005–32255	3.5	.82	−.09
3964.22	6249–31467	8.5	2.0	.30
3967.47	620–25818	.81	.19	−.72
3974.90	7645–32796	4.0	.94	−.03
3980.80	5762–30876	2.6	.61	−.21
3997.09	3800–28811	1.6	.39	−.41
3999.18	3801–28799	1.6	.38	−.41
4005.21	620–25580	2.7	.66	−.18
4005.70	620–25577	1.2	.29	−.54
4034.50	4453–29233	3.2	.78	−.11
4039.75	7645–32392	2.0	.50	−.30
4042.76	620–25349	6.1	1.5	.18
4047.62	620–25319	2.4	.60	−.22
4061.35	620–25235	.48	.12	−.92
4064.16	5991–30589	2.2	.54	−.27
4091.64	0–24433	.54	.14	−.87
4101.91	8118–32490	6.4	1.6	.21
4103.12	7326–31690	5.0	1.3	.10
4108.36	0–24334	.33	.084	−1.08
4116.88	3868–28151	.63	.16	−.79
4132.02	4275–28470	1.7	.44	−.36
4133.50	0–24186	.96	.25	−.61
4141.86	620–24757	.37	.094	−1.03
4146.61	7326–31435	1.4	.35	−.46
4153.97	0–24067	4.1	1.1	.02
4156.66	620–24671	2.0	.52	−.28
4160.95	0–24026	.31	.081	−1.09
4162.43	3801–27818	2.7	.69	−.16
4166.64	620–24613	.36	.093	−1.03
4169.06	8119–32098	4.7	1.2	.09
4186.96	4276–28153	2.3	.59	−.23
4191.94	0–23849	.24	.064	−1.20
4198.22	620–24433	.69	.18	−.74
4201.13	7645–31442	2.4	.63	−.20
4213.87	11308–35032	7.8	2.1	.32
4214.28	7645–31367	2.0	.54	−.27
4219.97	4275–27965	.78	.21	−.68
4222.36	3801–27478	3.6	.97	−.01
4231.67	7646–31270	5.3	1.4	.15
4236.04	620–24220	.80	.21	−.67
4246.26	0–23544	1.1	.29	−.53
4266.32	0–23433	.22	.061	−1.22
4267.94	6249–29672	.93	.25	−.60
4288.84	6249–29559	3.4	.94	−.03
4306.82	0–23212	.11	.030	−1.53
4313.13	3801–26979	1.4	.39	−.41
4335.73	0–23058	.47	.13	−.88
4354.55	3800–26758	1.2	.35	−.46
4355.75	620–23572	1.9	.54	−.27
4362.05	0–22919	1.6	.46	−.33
4371.76	8119–30986	2.2	.64	−.19
4372.76	0–22862	.25	.072	−1.14
4383.27	3801–26608	.47	.14	−.86
4393.60	0–22754	1.2	.34	−.47
4426.94	0–22583	.24	.070	−1.15
4440.74	3801–26313	.34	.10	−1.00
4469.32	0–22368	.18	.055	−1.26
4516.73	620–22754	.25	.077	−1.11
4551.98	620–22583	.17	.053	−1.27
4563.95	13127–35032	3.4	1.1	.02
4576.64	620–22464	.19	.060	−1.22
4620.23	6249–27887	3.7	1.2	.07
4631.62	0–21585	.68	.22	−.66
4663.75	620–22056	.18	.058	−1.24
4715.68	10208–31408	1.3	.45	−.35
4743.53	6249–27324	.45	.15	−.82
4756.80	620–21637	.50	.17	−.77
4768.66	620–21585	.12	.042	−1.38
4778.10	10347–31270	1.1	.38	−.41
4780.19	10081–30994	1.1	.36	−.44
4785.91	3868–24757	.25	.087	−1.06
4790.06	7645–28516	.70	.24	−.62
4810.90	3801–24581	.25	.085	−1.07
4842.48	620–21265	.15	.054	−1.27
4868.86	3801–24334	.23	.083	−1.08
4885.15	0–20464	.11	.041	−1.39
4910.35	6249–26608	.53	.19	−.72
4928.44	4276–24560	.29	.11	−.97
4933.06	3801–24067	.15	.054	−1.26
4955.78	7646–27818	.62	.23	−.64
4967.33	3801–23927	.25	.094	−1.03
5011.42	620–20569	.068	.026	−1.59
5027.38	0–19885	.44	.17	−.78
5063.77	3801–23544	.20	.078	−1.11
5088.29	0–19648	.050	.020	−1.71
5164.14	8119–27478	.85	.34	−.47
5272.00	7646–26608	.20	.083	−1.08
5280.38	0–18933	.15	.062	−1.21
5308.54	3801–22633	.20	.086	−1.07
5315.27	7646–26454	.22	.094	−1.03
5319.38	10081–28874	.43	.18	−.73
5329.26	0–18759	.084	.036	−1.45
5382.94	620–19192	.027	.012	−1.93
5385.54	13127–31690	.46	.20	−.69
5389.83	10347–28895	.39	.17	−.77
5400.90	10288–28798	.72	.32	−.50
5410.24	4276–22754	.073	.032	−1.50
5423.35	5762–24195	.084	.037	−1.43
5459.27	620–18933	.041	.018	−1.74
5496.43	4276–22464	.17	.078	−1.11
5500.69	620–18794	.035	.016	−1.80
5510.41	13127–31270	1.3	.59	−.23
5511.49	620–18759	.099	.045	−1.35
5513.39	7326–25458	.16	.073	−1.14
5531.26	7864–25938	.18	.082	−1.08
5557.87	620–18607	.048	.022	−1.65
5564.17	3801–21768	.30	.14	−.86
5573.07	10347–28286	.27	.12	−.91
5573.59	6249–24186	.087	.040	−1.39
5608.86	15720–33544	.89	.42	−.38
5610.89	6249–24067	.42	.20	−.70
5616.58	8119–25918	.16	.074	−1.13
5620.78	620–18406	.12	.055	−1.26
5621.51	3801–21585	.12	.057	−1.24
5632.47	10069–27818	.24	.11	−.94
5634.38	7646–25389	.29	.14	−.86
5636.78	7005–24741	.080	.038	−1.42
5640.30	5762–23486	.090	.043	−1.37
5648.38	10557–28256	.18	.085	−1.07
5658.26	3868–21536	.077	.037	−1.43
5669.42	620–18254	.057	.027	−1.56
5680.37	6249–23849	.098	.047	−1.32
5685.19	4453–22038	.065	.031	−1.50
5705.66	10819–28341	.23	.11	−.95
5709.49	7103–24613	.098	.048	−1.32
5716.87	4453–21941	.096	.047	−1.33
5722.23	13535–31005	.42	.21	−.68
5736.38	7006–24433	.19	.093	−1.03
5750.54	8856–26241	.26	.13	−.89
5758.14	0–17362	.038	.019	−1.73
5758.35	4276–21637	.050	.025	−1.60
5761.88	16244–33595	.61	.31	−.51
5763.59	7103–24449	.048	.024	−1.63
5763.66	7326–24671	.050	.025	−1.60
5765.41	8118–25458	.12	.060	−1.22
5767.43	5991–23325	.074	.037	−1.44
5771.05	6249–23572	.078	.039	−1.41
5777.77	12362–29665	.31	.16	−.80
5780.59	6249–23544	.47	.23	−.63
5796.51	17091–34338	.92	.46	−.34
5802.11	8119–25349	.41	.21	−.68
5805.20	5991–23212	.072	.036	−1.44
5813.83	5991–23187	.072	.036	−1.44
5814.41	3868–21062	.035	.018	−1.75
5816.79	10288–27475	.19	.097	−1.01
5819.01	7006–24186	.072	.037	−1.43
5836.03	10347–27478	.77	.39	−.41
5852.01	4453–21536	.050	.025	−1.59
5898.78	6249–23197	.13	.069	−1.16
5902.50	3868–20805	.085	.044	−1.35
5915.40	0–16900	.35	.18	−.74
5925.47	7646–24517	.20	.10	−.98
5929.33	3801–20662	.074	.039	−1.41
5933.82	620–17468	.028	.015	−1.83
5942.77	5762–22584	.058	.031	−1.52
5948.57	7646–24452	.12	.063	−1.20
5956.86	3868–20651	.074	.040	−1.40
5971.50	620–17362	.098	.052	−1.28
5976.32	3801–20529	.36	.19	−.71
5986.10	3868–20569	.10	.055	−1.26
5997.31	6249–22919	.28	.15	−.82
5997.96	4276–20943	.062	.034	−1.47
5999.41	3801–20464	.080	.043	−1.37
6000.19	11308–27969	.40	.22	−.67
6008.87	10254–26892	.31	.17	−.77
6010.86	10347–26979	.32	.17	−.76
6014.07	14845–31468	.89	.48	−.32
6016.73	5762–22378	.056	.030	−1.52
6017.57	6249–22862	.12	.068	−1.17
6019.19	4453–21062	.064	.035	−1.46
6028.13	3868–20452	.080	.044	−1.36
6035.54	7191–23755	.051	.028	−1.56
6039.60	8119–24671	.19	.10	−.99
6050.48	3868–20392	.032	.017	−1.76
6050.67	7326–23849	.070	.038	−1.42
6056.80	0–16506	.023	.013	−1.90
6057.07	6249–22754	.095	.052	−1.28
6062.30	4276–20766	.069	.038	−1.42
6077.29	620–17070	.15	.082	−1.08
6089.19	3801–20219	.054	.030	−1.53
6101.77	6249–22633	.11	.059	−1.23
6127.77	8119–24433	.14	.079	−1.10
6129.72	620–16930	.045	.026	−1.59
6132.61	11308–27609	.37	.21	−.67
6138.54	10685–26971	.14	.081	−1.09
6152.25	4275–20525	.033	.019	−1.73
6164.50	7326–23544	.058	.033	−1.48
6171.85	7646–23844	.31	.18	−.75
6175.38	4276–20464	.12	.070	−1.15
6215.37	3801–19885	.051	.029	−1.53
6234.30	4276–20312	.056	.033	−1.49
6246.53	5762–21766	.078	.046	−1.34
6268.66	8119–24067	.11	.067	−1.17
6292.03	8133–24022	.11	.067	−1.17
6293.32	620–16506	.030	.018	−1.74
6298.53	4275–20148	.062	.037	−1.43
6359.28	0–15721	.036	.022	−1.66
6372.43	3801–19489	.17	.10	−.99
6372.97	5762–21448	.026	.016	−1.79
6383.59	4453–20114	.027	.017	−1.78
6389.77	5991–21637	.11	.068	−1.17
6392.74	0–15638	.035	.022	−1.67
6395.42	0–15632	.11	.069	−1.16
6397.14	7006–22633	.11	.068	−1.16
6411.59	7326–22919	.060	.037	−1.43
6430.93	5991–21536	.038	.024	−1.63
6449.17	620–16122	.16	.099	−1.00
6465.00	7326–22790	.22	.14	−.86
6481.72	5762–21185	.020	.013	−1.89
6488.35	13535–28943	.24	.15	−.82
6503.62	4276–19648	.050	.032	−1.50
6518.94	6249–21585	.078	.050	−1.30
6520.98	8133–23464	.034	.022	−1.66
6526.08	7645–22964	.046	.029	−1.53
6527.04	5762–21079	.020	.013	−1.89
6542.98	10347–25626	.11	.073	−1.14
6552.75	7326–22583	.021	.014	−1.87
6555.01	3868–19119	.038	.025	−1.61
6582.78	7864–23051	.024	.015	−1.81
6601.39	7646–22790	.030	.019	−1.71
6603.98	7326–22464	.062	.041	−1.39
6620.52	620–15721	.037	.024	−1.61
6625.29	5762–20852	.048	.032	−1.50
6628.65	11677–26758	.11	.073	−1.14
6647.79	3800–18839	.024	.016	−1.79
6656.81	620–15638	.012	.0078	−2.11
6683.38	3801–18759	.054	.036	−1.44
6691.21	10686–25627	.058	.039	−1.41
6727.96	5762–20621	.019	.013	−1.90
6736.80	0–14839	.017	.012	−1.93
6754.93	8119–22919	.047	.032	−1.49
6768.64	7864–22634	.030	.020	−1.69
6780.62	8118–22862	.031	.021	−1.67
6782.70	3868–18607	.012	.0081	−2.09
6782.85	4453–19192	.013	.0093	−2.03
6790.30	7646–22368	.14	.096	−1.02
6812.98	4453–19127	.0067	.0046	−2.33
6813.75	11677–26349	.035	.024	−1.62
6813.81	11633–26305	.034	.024	−1.62
6818.29	3868–18531	.029	.020	−1.69
6820.76	4276–18933	.11	.078	−1.11
6824.45	7103–21753	.073	.051	−1.29
6826.93	0–14644	.17	.12	−.93
6832.71	7006–21637	.036	.025	−1.60
6846.25	6249–20852	.050	.035	−1.46
6869.07	19761–34315	2.7	1.9	.28
6887.74	8119–22633	.090	.064	−1.19
6902.55	4276–18759	.031	.022	−1.65
6915.31	5762–20219	.052	.037	−1.43
6917.05	3801–18254	.069	.050	−1.30
7015.70	8119–22368	.086	.064	−1.20
7030.69	620–14839	.016	.011	−1.94
7033.84	10347–24560	.30	.22	−.66
7074.78	4276–18406	.15	.11	−.96
7101.61	4453–18531	.18	.14	−.86
7128.89	620–14644	.088	.067	−1.18
7130.05	5762–19783	.041	.031	−1.51
7147.87	10347–24334	.40	.31	−.51
7164.87	4453–18406	.059	.046	−1.34
7172.10	14845–28784	.32	.25	−.61
7194.63	10685–24581	.24	.19	−.72
7196.66	10081–23972	.11	.083	−1.08
7205.42	10686–24560	.12	.095	−1.02
7210.28	6249–20114	.071	.055	−1.26
7254.44	7645–21426	.21	.17	−.78
7371.95	5991–19552	.040	.032	−1.49
7390.99	10208–23734	.10	.085	−1.07
7392.11	13127–26652	.20	.17	−.78
7396.98	4453–17969	.083	.068	−1.17
7416.57	10081–23560	.10	.082	−1.08
7425.50	0–13463	.050	.041	−1.39
7517.39	16040–29339	.38	.32	−.49
7528.70	19103–32381	1.5	1.3	.11
7533.91	3801–17070	.23	.20	−.71
7550.23	10685–23926	.28	.24	−.63
7590.52	5762–18933	.036	.031	−1.51
7595.04	10686–23849	.11	.095	−1.02
7600.27	16040–29194	.30	.26	−.59
7609.16	7326–20464	.10	.088	−1.06
7619.34	7646–20766	.19	.17	−.78
7631.72	3801–16900	.056	.049	−1.31
7634.74	7326–20420	.080	.070	−1.15
7639.54	4276–17362	.075	.066	−1.18
7653.62	8878–21940	.057	.050	−1.30
7748.19	16040–28943	.57	.51	−.29
7754.18	7326–20219	.078	.070	−1.15
7759.87	7646–20529	.084	.076	−1.12
7761.84	7006–19885	.11	.10	−.99
7784.13	620–13463	.031	.028	−1.55
7816.32	13128–25918	.29	.26	−.58
7832.02	14845–27609	.19	.17	−.77
7835.71	5991–18749	.018	.016	−1.79
7844.71	16040–28784	.70	.64	−.19
7868.75	3801–16506	.063	.059	−1.23
7875.36	11308–24002	.35	.33	−.48
7881.94	6249–18933	.41	.38	−.42
7900.43	4276–16930	.047	.044	−1.36
7904.28	8119–20766	.079	.074	−1.13
7907.96	7006–19648	.061	.057	−1.24
7918.80	4276–16900	.047	.044	−1.36
7959.96	7326–19885	.065	.062	−1.21
7963.96	10081–22634	.26	.25	−.61
7970.46	8119–20662	.22	.21	−.68
7975.08	11308–23843	.23	.22	−.66
7975.47	18933–31468	.46	.44	−.36
7991.30	6249–18759	.050	.048	−1.32
7998.60	13128–25627	.35	.33	−.48
8012.96	4453–16930	.017	.016	−1.80
8019.38	7006–19472	.051	.049	−1.31
8034.79	10347–22790	.18	.18	−.76
8055.61	8119–20529	.076	.074	−1.13
8065.47	10069–22464	.17	.16	−.78
8065.84	3800–16195	.014	.014	−1.86
8097.62	8118–20464	.11	.11	−.98
8126.23	10081–22383	.10	.099	−1.00
8137.21	10347–22633	.12	.12	−.91
8153.71	13128–25389	.23	.23	−.63
8174.30	4276–16506	.077	.077	−1.11
8175.84	16040–28268	.45	.45	−.34
8230.83	5762–17908	.043	.043	−1.36
8240.51	5762–17894	.021	.022	−1.66
8262.05	8119–20219	.21	.21	−.67
8310.69	11457–23486	.18	.18	−.74
8318.34	4275–16294	.075	.077	−1.11
8329.74	7645–19647	.064	.067	−1.17
8346.74	5991–17969	.078	.082	−1.09
8357.07	3868–15831	.027	.028	−1.55
8381.86	7006–18933	.16	.17	−.78
8387.19	4275–16195	.034	.035	−1.45
8389.16	5991–17908	.078	.082	−1.09
8441.20	7646–19489	.16	.17	−.78
8445.35	3801–15638	.15	.16	−.80
8450.02	3801–15632	.074	.080	−1.10
8496.09	8119–19885	.079	.086	−1.07
8504.53	11457–23212	.17	.18	−.74
8540.19	5762–17468	.072	.078	−1.11
8542.32	10254–21958	.064	.070	−1.16
8557.32	10686–22368	.14	.15	−.81
8567.71	4453–16122	.034	.037	−1.43
8570.51	8119–19783	.17	.19	−.73
8574.59	6249–17908	.051	.056	−1.25
8607.96	0–11614	.13	.14	−.84
8641.12	16040–27609	.59	.66	−.18
8659.57	10208–21753	.12	.14	−.86
8691.28	0–11502	.037	.042	−1.37
8710.77	5991–17468	.12	.13	−.88
8753.68	10347–21768	.31	.36	−.44
8757.77	620–12035	.059	.068	−1.17
8816.56	7191–18530	.075	.088	−1.06

**Table 2:** Upper limits to lifetimes for levels in U I

Level cm^−1^	*J*	*g*	Lifetime *ns*	*n*
16900.385	7	15	330.	3
18932.762	5	11	120.	6
19885.513	7	15	200.	5
21584.692	6	13	130.	4
22754.057	6	13	81.	4
22918.552	7	15	75.	4
23543.504	7	15	83.	4
23572.082	6	13	66.	2
24066.562	7	15	31.	4
24433.256	6	13	83.	4
24671.385	6	13	58.	3
25319.272	5	11	26.	2
25348.976	6	13	9.5	3
25462.659	6	13	66.	1
25580.748	6	13	47.	1
25672.464	7	15	49.	1
25825.562	6	13	14.	1
25938.231	6	13	30.	2
26225.568	6	13	9.8	1
26550.427	6	13	65.	1
26920.718	5	11	23.	2
27477.553	8	17	32.	3
27615.796	6	13	35.	2
27818.491	8	17	48.	3
27886.992	7	15	7.3	2
27938.049	5	11	42.	2
27941.251	6	13	14.	2
28053.058	6	13	24.	2
28067.646	5	11	17.	1
28118.841	7	15	39.	1
28444.515	5	11	11.	2
28470.179	6	13	28.	3
28503.449	5	11	24.	1
28562.631	5	11	29.	1
28650.295	5	11	4.5	2
28811.960	6	13	33.	3
28840.936	5	11	27.	1
28874.930	6	13	40.	3
29099.578	5	11	28.	1
29232.652	5	11	20.	2
29459.910	4	9	16.	1
29837.646	7	15	7.7	1
30107.096	4	9	12.	2
30747.892	7	15	26.	1
30875.581	6	13	11.	2
30986.296	8	17	27.	2
30993.006	5	11	12.	1
31178.758	8	17	16.	1
31270.334	9	19	25.	3
31279.130	8	17	12.	1
31435.401	7	15	23.	3
31467.623	6	13	15.	1
31649.690	6	13	11.	2
31871.569	6	13	15.	2
32098.168	7	15	15.	3
32412.827	6	13	7.7	1
32495.736	5	11	6.9	2
32604.071	5	11	9.7	1
32648.876	5	11	9.9	2
32742.570	5	11	6.6	1
33570.669	7	15	13.	1
33639.564	6	13	9.6	2
34046.448	3	7	6.5	2
34315.658	8	17	13.	2
34486.486	5	11	10.	2
